# Data supporting chitosan facilitates structure formation of the salivary gland by regulating the basement membrane components

**DOI:** 10.1016/j.dib.2015.07.006

**Published:** 2015-07-17

**Authors:** Ya-Chuan Hsiao, Tsung-Lin Yang

**Affiliations:** a Department of Otolaryngology, National Taiwan University Hospital and College of Medicine, Taipei, Taiwan; b Department of Ophthalmology, Zhongxing Branch, Taipei City Hospital, Taipei, Taiwan; c Department of Ophthalmology, College of Medicine, National Yang-Ming University, Taipei, Taiwan; d Research Center for Developmental Biology and Regenerative Medicine, National Taiwan University, Taipei, Taiwan

**Keywords:** Salivary glands, Chitosan, Basement membrane, Morphogenesis

## Abstract

To investigate the role of basement membrane (BM) in chitosan-mediated morphogenesis of the salivary glands, the embryonic submandibular gland (SMG) experimental model was used. Chitosan promotes branching at distinct stages in SMG morphogenesis. When enzymes such as type IV collagenase, dispase, and cathepsin B were used to digest the BM components, the morphogenetic effect mediated by chitosan disappeared. Immunofluorescence revealed that the corresponding receptors for BM components, including CD49c, CD49f, CD29, and dystroglycan, were locally enriched at the epithelial–mesenchymal junction around BM areas. The functional roles of laminin α1 and α5 in SMG branching were explored via siRNA knockdown, and suppression was confirmed at both the RNA and protein levels (Yang and Hsiao, Biomaterials, http://dx.doi.org/10.1016/j.biomaterials.2015.06.028, 2015). This data article demonstrates the experimental approaches to investigate the role of basement membrane in the structure formation of the salivary gland engineered by biomaterials.

**Specifications Table**

**Table:** 

Subject area	Biology
More specific subject area	Chitosan mediated morphogenetic effect of the salivary gland structure formation
Type of data	Figures and Charts
How data was acquired	An ex vivo culture of submandibular gland (SMG) explants was used. Enzymes were applied to digest away basement membrane (BM) components. The spatial distribution of BM receptors was demonstrated and analyzed via immunofluorescence staining. siRNA-mediated suppression of BM components was confirmed by qPCR and Western blotting
Data format	Raw and analyzed Data
Experimental factors	SMG explants were cultured in a chitosan-containing system to induce structure formation. Explants were then treated with specific reagents and harvested for molecular and cytological analyses
Experimental features	Image recordings were used for quantitative analyses, including branching numbers and immunofluorescence intensity. Quantitative PCR and Western blotting were performed and analyzed
Data source location	The National Taiwan University, Taipei, Taiwan
Data accessibility	Data is available with this article

**Value of the data**

• This data demonstrated the potential of using a standard experimental model of embryonic submandibular glands (SMGs) to investigate the morphogenetic effects of biomaterials
• Treatments of digesting enzyme and siRNA toward BM components render the investigation of BM function in salivary gland morphogenesis feasible.
• The spatial and temporal change of the BM components and corresponding receptors during the structure formation of the salivary glands could be explored by immunofluorescence settings and analyses.

## 1 Data, experimental design, materials and methods

The information and data presented in this data article demonstrated the experimental approaches to investigate the role of basement membrane in chitosan-mediated morphogenesis of the salivary gland.

## 2 Materials and methods

### 2.1 Preparation of the chitosan-containing system

The chitosan-containing system was established using a water-soluble form of chitosan. A 2 wt% (w/v) chitosan solution was prepared by dissolving chitosan (Sigma-Aldrich Chemical Co. St. Louis, MO, USA) in 1 M acetic acid. The solution was subsequently mixed with the standard culture medium used for SMG explant culture, neutralized with sodium hydroxide, and prepared at the indicated concentrations as previously described [2,3]. Mock medium was prepared in the same way as the chitosan-containing medium by adding the same amount of acetic acid and sodium hydroxide without chitosan. The mock and control media had similar effects on SMG without significant differences [4]. The control medium was therefore used for comparison in following assays.

### 2.2 Ex vivo explant culture of the submandibular gland (SMG)

Animal protocols were approved by the Animal Care and Use Committee of the National Taiwan University and were in accordance with the guidelines. E12.5 and E13 submandibular glands (SMGs) retrieved from ICR mice were used for explant culture, and the protocol followed the methods described previously [5]. Cultured SMG explants were photographed and measured at the indicated time-points. SMG branching was quantified as the fold-change in branch number between the chitosan and control groups. Each experiment was repeated at least three times for comparison.

### 2.3 Explant culture with enzyme treatment

The SMG explants were cultured as above. To evaluate the effect of collagenase on the branch-promoting effect of chitosan, distinct types of commercially available collagenase (Sigma-Aldrich, St Louis, MO, USA) were used, including collagenase (C0130) 0.1%; type I collagenase (C2674), 0.5 mg/ml; and type II collagenase (C6885), 1 mg/ml. Type IV collagenase was from Worthington (CLS-4), and was used at concentrations of 2, 20, 100, 200 U/ml. A commercial version of dispase (BD Bioscience, San Jose, CA, USA) was used at concentrations of 0.016, 0.16, 0.8, 1.6 U/ml [4]. Cathepsin B (Sigma, C6286) was used at the following concentrations of 0.002, 0.005, 0.01 U/ml.

### 2.4 Immunofluorescence imaging and quantitative analysis of intensity

Immunofluorescence staining was carried out as described previously [6]. Fluorescence expression was photographed and merged by confocal microscopy (Leica SP-5). For quantitative analysis of immunofluorescence intensity, fluorescence images from at least 3 SMG explants were analyzed using NIH Image J software based on the serial sections of confocal images obtained from individual SMG explants. At least 6 regions within each section were used for quantitative analysis of the fluorescence intensity. Total fluorescence was calculated and corrected for background fluorescence using published equations [7]. The regions included for analysis centered on the basement membrane (BM), the intracellular area below the BM, and the extracellular area outside the BM [2].

### 2.5 Confirmation of siRNA effect on RNA and protein levels

To confirm the suppression effect of siRNA on SMG branching mediated by chitosan, SMG explants treated with specific and scrambled siRNA were harvested for RNA and protein extraction. Quantitative PCR was used to confirm RNA suppression. For quantitative PCR, cDNA synthesis was performed using the RevertAid First Strand cDNA Synthesis Kit (Thermo). SYBR Green Supermix (Bio-Rad) was used for quantitative PCR (Bio-Rad iCycler MyiQ Real Time thermocycler). Primers specific to laminin α1 and laminin α5 were used [1]. Gene expression was normalized to ribosomal protein S29 expression Relative expression was determined by melting-curve analysis and presented as the fold-change (Bio-Rad IQ5). Western-blot analysis was used to detect protein suppression. Proteins extracted from SMG explants from different groups were separated by SDS-PAGE, (Bio-Rad, CA) and detected using specific antibodies (Novus Biologicals). Blots were developed using a Pierce BCA protein assay kit (Thermo Scientific) and analyzed on a chemiluminescent imager (UVP BioSpectrum 600). SuperSignal^®^ West Femto Maximum Sensitivity Substrate (Thermo Scientific) was used to detect chemiluminescence. The results were quantitated and compared.

## 3 Data

### 3.1 Chitosan facilitates branching morphogenesis of the salivary glands

**Fig. 1:**
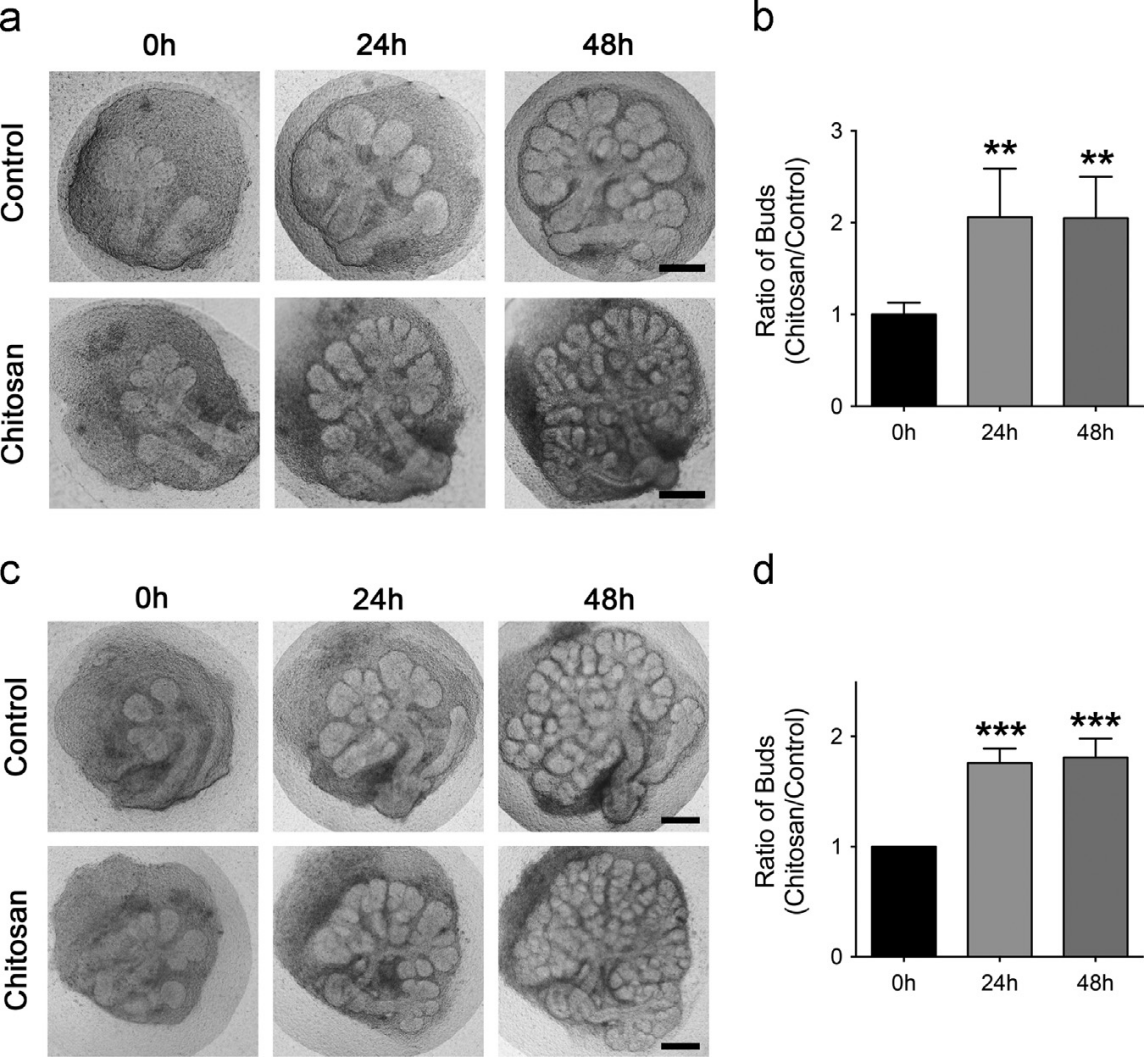
Branching structure formation of SMG explants in the chitosan-containing system. (a) E12.5 SMG explants cultured in the control (Cont) and chitosan (Chi) groups for 48 h. (b) Quantitative analyses of branches at the indicated time-points expressed as the ratio between control and chitosan groups. (c) E13 SMG explants cultured in the control (Cont) and chitosan (Chi) groups for 48 h. (d) Quantitative statistics of branch formation at the indicated time-points expressed as the ratio between control and chitosan groups. Scale bars in (a, c): 100 μm; data in (b and d) represent as means±SD. (*n*≥3). Student’s *t*-test was used to calculate *p*-values. ^**^p<0.001, ^***^p<0.0001.

Ex vivo culture of embryonic SMG explants, a standard model for studying branching morphogenesis, was used to investigate the morphogenetic effect of chitosan [5,8,9]. When SMG explants were harvested from E12.5 embryos, branching morphogenesis occurred in the following 48 h of culture. When chitosan was added to the culture system, SMG branching was promoted (Fig. 1a). The increase in the SMG branch number was significantly greater than that in the control group, at both 24 and 48 h (Fig. 1b). When the SMG explants were harvested from E13 embryos, an enhanced morphogenetic effect mediated by chitosan was also observed (Fig. 1c). SMG explants cultured in the chitosan-containing system had greater numbers of branches than control explants at the indicated time-points during the culture periods (Fig. 1d) [10,11].

### 3.2 The effect of enzyme treatment on SMG branching mediated by chitosan

**Fig. 2:**
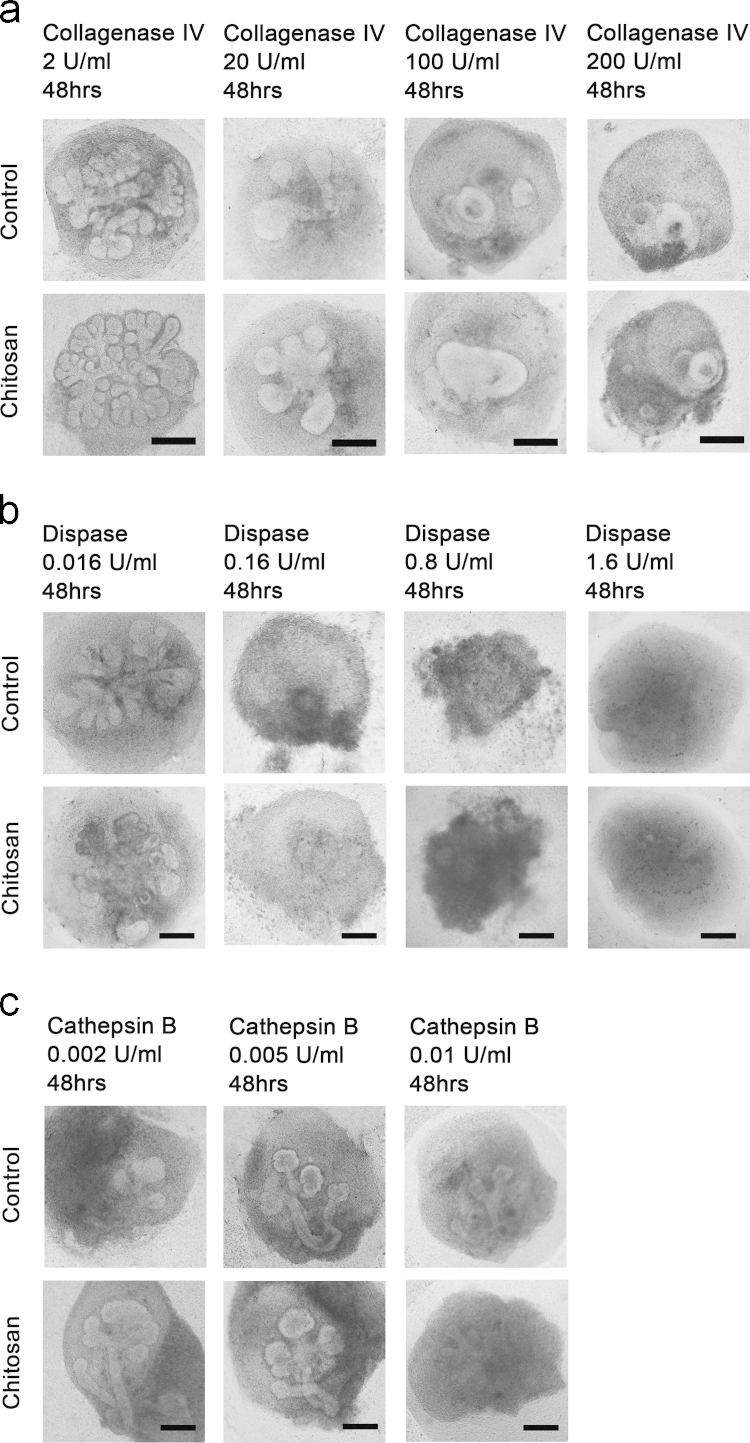
The effect of enzyme treatment on SMG branching mediated by chitosan. SMG explants cultured in the control and chitosan groups were treated with different concentrations of (a) type IV collagenase, (b) dispase, (c) cathepsin B, for 48 h. Scale bars: 100 μm.

To explore the role of BM components in the morphogenetic effect of chitosan, the cultured SMG explants were treated with different enzymes capable of digesting BM components. Here, collagenases with broad effects were applied, including *Clostridial,* type I, and type II collagenase. *Clostridial* collagenase is a mixture of clostripain and a neutral protease, which synergistically degrade collagen. Type I collagenase has the balance effects among collagenase, caseinase, clostripain and tryptic activities. Type II collagenase has a high level of clostripain activity. When these collagenases were applied, no branching morphogenesis was observed in both the control and chitosan groups (Fig. 1a in Refs. [1,12]). SMG branching still occurred when SMG explants were cultured with type IV collagenase but was seriously affected when the dose of type IV collagenase was increased (Fig. 2a) [1]. Dispase is a neutral protease that functions as a fibronectinase and type IV collagenase to degrade BM [13]. When dispase was employed, SMG morphogenesis was severely impaired (Fig. 2b). When a low concentration of dispase was used, branching developed in both groups, with no differences in branch numbers. At a high dose of dispase, no branching structures remained (Fig. 2b) [1]. Furthermore, darkening regions were observed around SMG explants, indicating the detrimental effect of high doses of dispase on the viability of SMG explants (Fig. 2b) [14]. Moreover, laminin (LN) serves as the crux to generate BM networks. When cathepsin B, an enzyme capable of digesting LN, was applied, SMG structure formation was affected (Fig. 2c) [15]. Branching still occurred in both groups but with lower numbers. Branching was still greater in the chitosan group than in the control group when 0.002 U/ml cathepsin B was added (Fig. 2c). Notably, the difference between the two groups decreased with increasing doses of cathepsin B [1]. There was no difference in branching between the two groups with 0.005 U/ml cathespin B (Fig. 2c). The SMG branching phenotypes were devastated when the dose increased to 0.01 U/ml (Fig. 2c). Accordingly, the data showed that the morphogenetic effect of chitosan progressively disappeared with increasing doses of enzymes that specifically digest BM components.

### 3.3 Spatial expression pattern of BM receptors in the SMG explants cultured with chitosan

**Fig. 3:**
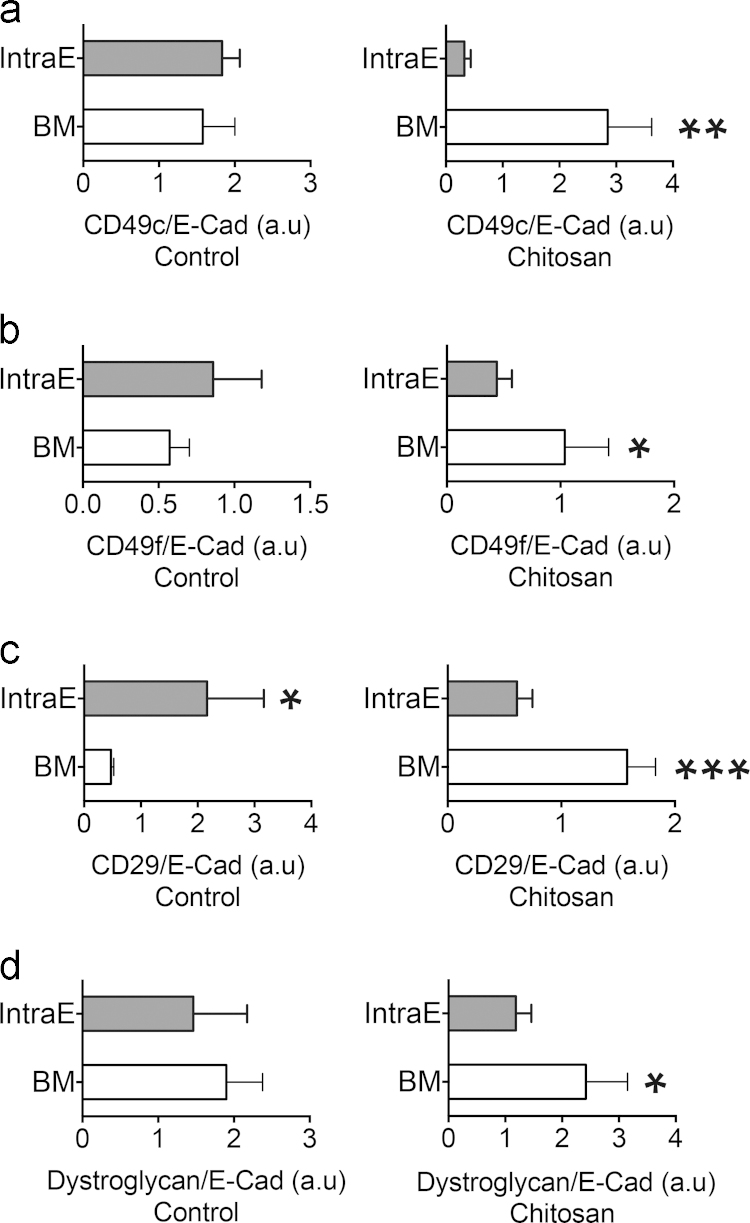
Quantitative analysis of the spatial expression pattern of BM receptors in SMG explants cultured with chitosan. The fluorescence intensity of BM receptors in the control (Cont) and chitosan (Chi) groups were graphed and analyzed. Expression was normalized to E-Cadherin. (a) CD49c, (b) CD49f, (c) CD29, (d) dystroglycan. Student’s *t*-test was used to calculate *p*-values. ^⁎^*p*<0.01, ^⁎^^⁎^*p*<0.001, ^⁎^^⁎^^⁎^*p*<0.0001.

The cellular locations of BM receptors are relevant to signaling transmission during morphogenesis [16]. Therefore, the expression of BM receptors was categorized into intraepithelial (IntraE) and BM groups. CD49c immunostaining was located to both intraE and BM areas. No difference was observed between these two categories in the control group, whereas the ratio of BM was greater than that of intraE in the chitosan group (Fig. 3a). A similar expression pattern was found for CD49f. In the control group, the expression levels were not different in either category. However, a greater ratio was found in BM rather than IntraE in the chitosan group (Fig. 3b). For CD29, a higher ratio was found in IntraE than in BM in the control group. Conversely, the ratio of BM was greater than IntraE in the chitosan group (Fig. 3c). When IntraE and BM were compared based on dystroglycan expression, a significant increase in BM was noted in the chitosan group (Fig. 3d). The ratio of intensity between the chitosan and control groups was greater in the BM category for all receptors tested. Taken together, these data demonstrated that BM receptors were recruited around the BM area in the chitosan-culture environment.

### 3.4 Blocking effect of siRNA on the SMG explants cultured with chitosan

**Fig. 4:**
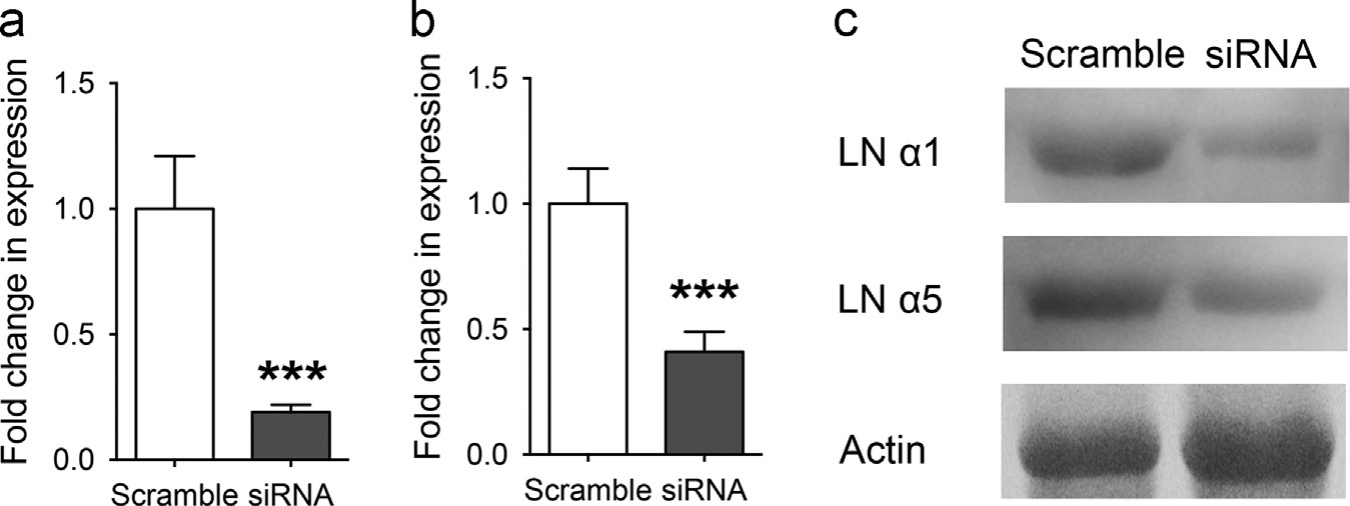
Suppressive effect of BM components siRNA on the SMG explants cultured with chitosan. qPCR was used to confirm the suppression of (a) *lama1*, and (b) *lama5* by siRNA. (c) The suppression of laminin α1 and α5 in the protein levels was confirmed by Western blot (LN: laminin; Student’s *t*-test was used to calculate *p*-values. ^⁎⁎⁎^*p*<0.0001).

Laminin is the principal molecule that constitutes the BM framework. Laminin was found to be expressed at high levels in the presence of chitosan and was locally concentrated at the epithelial-mesenchymal junction to facilitate SMG branching [1]. To test the functional roles of laminin in chitosan-mediated SMG morphogenesis, siRNA knockdown on laminin α1 and laminin α5 was performed in cultured SMG explants [1]. Quantitative PCR was used to confirm the suppression of laminin α1 and laminin α5 RNA. Laminin α1 and laminin α5 RNA expression decreased in SMG explants cultured with siRNA (Fig. 4a and b). Western blotting was also used to confirm protein suppression, and the protein intensities were decreased in the siRNA-treated group (Fig. 4c). These results confirmed the effect of siRNA treatment in cultured SMG explants.
